# Intermittent Proton Pump Inhibitor Therapy in Low-Risk Non-Variceal Upper Gastrointestinal Bleeding May Be Significantly Cost-Saving

**DOI:** 10.3390/medicines10070044

**Published:** 2023-07-20

**Authors:** Yang Lei, Jennifer Halasz, Kerri L. Novak, Stephen E. Congly

**Affiliations:** 1 Division of Gastroenterology and Hepatology, Department of Medicine, Cumming School of Medicine, University of Calgary, Calgary, AB T2N 4Z6, Canada; yang.lei@ucalgary.ca (Y.L.); halasz@ualberta.ca (J.H.); knovak@ucalgary.ca (K.L.N.); 2 O’Brien Institute of Public Health, University of Calgary, Calgary, AB T2N 4Z6, Canada

**Keywords:** proton pump inhibitor, non-variceal GI bleed, cost-saving, health economics, resource utilization

## Abstract

Background: High-dose proton pump inhibitor (PPI) therapy, given either intermittently or continuously for non-variceal upper gastrointestinal bleeding (NV-UGIB), is efficacious. Using intermittent PPI for low-risk patients may be cost-saving. Our objective was to estimate the annual cost savings if all low-risk NV-UGIB patients received intermittent PPI therapy. Methods: Patients who presented to hospital in Calgary, Alberta, who received a PPI for NV-UGIB from July 2015 to March 2017 were identified using ICD-10 codes. Patients were stratified into no endoscopy, high-risk, and low-risk lesion groups and further subdivided into no PPI, oral PPI, intermittent intravenous (IV), and continuous IV subgroups. Average length of stay (LOS) in each subgroup and costs were calculated. Results: We identified 4141 patients with NV-UGIBs, (median age 61, 57.4% male). One-thousand two-hundred and thirty-one low-risk patients received continuous IV PPI, with an average LOS of 6.8 days (95% CI 6.2–7.3) versus 4.9 days (95% CI 3.9–5.9) for intermittent IV patients. If continuous IV PPI patients instead received intermittent IV PPI, 3852 patient days and CAD 11,714,390 (2017 CAD)/year could be saved. Conclusions: Using real-world administrative data, we demonstrate that a sizable portion of low-risk patients with NV-UGIB who were given continuous IV PPI if switched to intermittent IV therapy could generate significant potential cost savings.

## 1. Introduction

Upper gastrointestinal bleeding (UGIB) is a common reason for gastrointestinal-related hospital admissions and is a major cause for morbidity, mortality, and cost. Current guidelines for treatment of non-variceal UGIB include fluid resuscitation, gastric mucosa-protecting medications, and endoscopy [1]. Proton pump inhibitors (PPI) are routinely used in clinical practice, both before and after endoscopy, as standard of care [2]. Pre-endoscopy PPI, largely given intravenously (IV), reduces stigmata of high-risk bleeding at endoscopy and the need for endoscopic therapy but has no effect on rebleeding, surgery, or mortality [3,4]. Post-endoscopy PPI for 72 h shows reduced bleeding and surgery rates in patients who received endoscopic hemostasis therapy [5].

The optimal use of PPI is controversial. Previous systematic reviews [6,7,8,9] show no difference in mortality between intermittent versus continuous PPI for 72 h post-endoscopy. More recently, the International Consensus Group on Upper GI Bleeding guidelines demonstrate a mortality benefit for IV continuous PPI but not for other PPI doses [8,10], whereas the updated American College of Gastroenterology Guidelines from 2021 do not prioritize continuous IV PPI over less intensive doses [11]. Ambulatory 72 h pH monitoring shows all high-dose PPI, regardless of which specific one, regardless of administration route (IV or PO), can maintain gastric pH above 6, compared to the mean pH without PPI use of 2.04 [12,13,14].

Modelling suggests that PPI pre-endoscopy is more effective and more costly than placebo in North America [15]. Data regarding savings with PPI have been variable; a budget impact analysis showed very modest savings with alternative PPI regimens compared to the standard high dose IV continuous PPI pre- and post-endoscopy [16]. A trainee-led initiative in Chicago demonstrated a 35% reduction in inappropriate PPI prescriptions; using intermittent PPI instead of PPI infusion led to cost savings estimated at USD 277 in 2017 per patient per day, yielding a minimum USD 121,000 over 9 months of savings in pharmacy costs [17]. Given these findings, our objective was to analyze potential economic effects in the Calgary Zone of Alberta Health Services, using administrative and clinical data, if all IV continuous PPI therapy were given as IV intermittent instead for patients admitted with suspected non-variceal upper GI bleeds (NV-UGIB).

## 2. Materials and Methods

### 2.1. Cohort Analysis

All patient visits that used an UGIB order set to prescribe PPI in the shared electronic order entry system from July 2015 to March 2017 at four adult hospitals in Calgary, Alberta, were obtained in de-identified form. The study dates were chosen to coincide with the baseline data collection dates for a separate quality improvement project. Using previously validated ICD-10 codes, as diagnoses in the administrative databases are classified using the ICD-10 system (Table S1) [18,19,20], we identified patients with NV-UGIB. The modality of PPI administration was determined at the discretion of the treating physician.

Patients were classified into three categories by Canadian Classification of Health Interventions (CCI) procedure codes (Table S2): (a) high-risk UGIB requiring intervention, defined as esophagogastroduodenoscopy (EGD) or interventional radiology (IR), (b) low-risk UGIB requiring EGD with biopsy or inspection, or (c) no EGD. Patients with procedure codes for variceal banding or gluing of gastric varices were excluded. Next, each risk category was further sorted by PO PPI (any frequency), intermittent IV (pantoprazole 40 mg IV either daily or bid), continuous IV (pantoprazole 80 mg IV or 8 mg/h IV), or no PPI. Each category was analyzed for demographic factors, presenting heart rate (HR), blood pressure (BP), hemoglobin (HGB), percent transfused, and percent admitted to hospital. Length of stay (LOS) was calculated both as an average and as a median. The proportion of patients on prolonged PPI therapy (more than 72 h) was also calculated for high-risk and low-risk groups. Statistical differences between categorical categories were determined through the use of a Kruskal–Wallis test; a *p*-value of <0.05 was considered statistically significant. Data analysis was done using Microsoft Excel v16.75 and SPSS v28.

### 2.2. Costing

We used the Canadian Institute of Health Information (CIHI) Case Mixed Groups+ (CMG+) methodology to estimate the cost of hospitalization which is a form of diagnosis related grouping [21] similar to diagnosis-related groups (DRG). The CMG+ calculates a resource intensity weight (RIW) based on nursing (inpatient, outpatient, operating room, and recovery), clinical lab, medical imaging, other professional services (e.g., laboratory, nutrition, physiotherapy, and social work), and indirect costs (e.g., staff transport, housekeeping, laundry services, and health records). The Alberta Government Interactive Health Data Application combines this RIW with Alberta-specific costs of a standard hospital stay (CSHS) to generate Alberta zone specific Inpatient Care Case Costs [22]. We utilized CMG+ code 254 (Gastrointestinal Hemorrhage) looking at all cases of severity in the Calgary zone for the 2016/2017 fiscal year. The average cost per hospitalization was CAD 7981.45 with an average length of stay of 6.22 days; the average cost per day was CAD 1283.21. Physician fees were calculated from the Alberta Health Care Insurance Plan schedule of medical benefits for 2017 [23]; we assumed an attending gastroenterologist saw the patient in consultation (CAD 186.95), potentially performed an endoscopy (CAD 113.19), and spent 20 min for subsequent daily care of the patient (CAD 104.90/day). Local data were used for drug costs for all modalities of IV PPI given. The cost per dose of 40 mg IV pantoprazole was CAD 2.20. A 72 h course of IV + bolus pantoprazole requires 16.4 doses (CAD 36.08) while IV intermittent dosing requires 6 doses (CAD 13.20). Twice-daily oral PPI was approximately CAD 0.40/day. After completing IV PPI courses, patients were assumed to be started on twice-oral PPI. All costs were in 2017 Canadian dollars.

This study was approved by the Conjoint Health Research Ethics Board at the University of Calgary (ID # REB16-0802).

## 3. Results

Our initial data capture had 5625 patient visits, of which 1484 were excluded as they did not have codes reflecting a NV-UGIB. Of the remaining 4141 patient visits, 410 (9.9%) were high-risk; 1403 (33.9%) were low-risk; and 2241 (54.1%) did not undergo EGD in the same admission (Table 1).

Overall, most patients who presented were male; patients who received endoscopy had a median age of 65. The no EGD group was younger and had a higher proportion of females than the low-risk and high-risk groups; full demographic details are in Table 1. Patients who did not receive an EGD had lower acuity (higher blood pressure, higher hemoglobin, and lower rate of transfusion and admission) than the low-risk group, which had more favourable clinical data than the high-risk group. Differences between treatment strategies were seen most in the high-risk group; the continuous IV subgroup had lower presenting hemoglobin (by around 10 g/L) and blood pressure (by around 10 mm Hg) compared to the intermittent IV subgroup (Table 1). The differences between the three groups regarding age, blood pressure, and hemoglobin were all statistically significant (*p* < 0.05) except for age, heart rate, and length of stay between the high-risk and low-risk groups.

**Figure 1:**
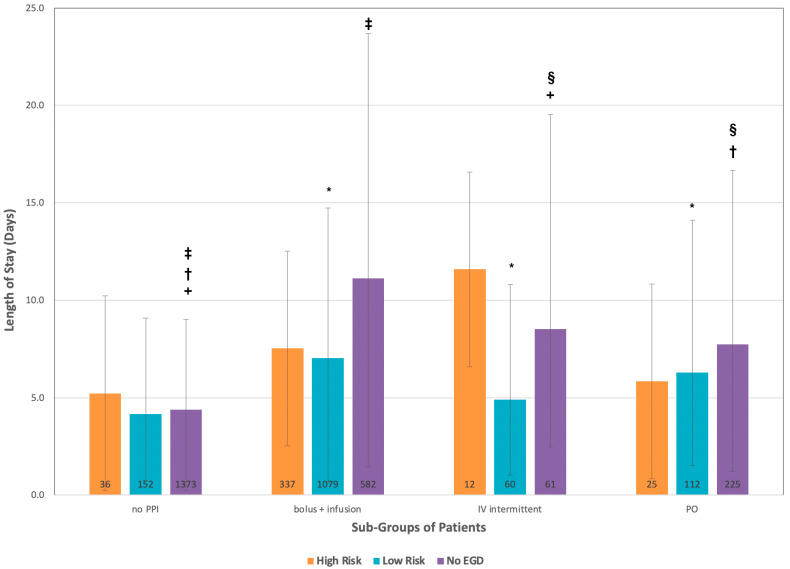
Length of stay stratified by risk of bleeding and treatment. Numbers of patients in each group reported at base of graph; 95% confidence intervals reported in whiskers. PPI = proton pump inhibitor. PO: Per os. EGD: Esophagogastroduodenoscopy. There were statistically significant differences in the low-risk population between the no PPI and each of the PPI groups (* *p* < 0.001) and in the no EGD population between the no PPI and intermittent infusion group(+ *p* < 0.001, no PPI and oral PPI, († *p* < 0.001), no PPI and continuous infusion group(‡ *p* < 0.001) and the oral PPI and intermittent infusion groups (§ *p* = 0.003).

For LOS, there were minimal difference in the median LOS (Figure 1). For average LOS (with 95% confidence intervals in Table 2), the high-risk group stayed 7.4 days, with the intermittent IV subgroup having longer LOS (11.6 days) than the continuous IV subgroup (7.5 days). The differences in LOS were statistically significant between the no EGD group and both the low-risk and high-risk groups (*p* < 0.001), but there was no statistically significant difference between the high-risk and low-risk groups.

In subgroup analysis, the average LOS of stay for the high-risk group was 7.4 days with no statistical difference in the LOS between any of the treatment strategies. For the low-risk group, the average LOS was 6.6 days, with the intermittent IV subgroup (4.9 days) being less than the continuous IV subgroup (6.8 days) with no significant difference between any of the PPI strategies although there was a statistically significant difference between the no PPI group and each of the PPI groups (*p* < 0.001). For patients who did not receive an EGD, the average LOS was 7.9 days, with patients receiving intermittent IV PPI (8.5 days) having shorter LOS than patients receiving continuous IV PPI (11.1 days). Statistically significant differences were seen between all groups (*p* ≤ 0.003) except for between the oral PPI and intermittent IV PPI groups and the continuous IV PPI and intermittent IV PPI groups. Thus, the groups of particular interest are the low-risk patient who received continuous IV PPI (representing 30% of all NV-UGIB visits) and patients that did not undergo EGD who received continuous IV PPI, representing 14.35% of all patients.

Based on our costing estimates, the 4141 patients admitted with upper GI bleeding cost the health care system approximately CAD 38 million dollars (Table 2). Patients who received intermittent PPI in the higher-risk group had the highest costs related to the length of stay. Conversely, in the low-risk group, patients who received intermittent PPI cost CAD 2660 less per admission less than those who had continuous PPI dosing with no difference in outcomes. Similarly, patients in the no EGD group cost CAD 3630 less per admission. Given the mixed evidence and guidance around PPI therapy for high-risk patients, to be conservative they were excluded (*n* = 410) from the cost savings calculation. If all patients who received continuous IV PPI in the low-risk and no EGD groups received intermittent IV PPI dosing instead over the study period, there could potentially be 1.9 days of LOS saved for 1231 low-risk patients (2338.9 days in total, CAD 6.2 million in savings) and 2.6 days of LOS saved for 582 no EGD patients (1513.2 days in total, CAD 3.5 million in savings). This corresponds to total potential savings of CAD 11.7 million over 21 months or CAD 6.7 million per year equivalent to 30% savings.

## 4. Discussion

In this analysis of 4141 patient visits over nearly two years in the Calgary Zone, we determined that 88% of patients did not require endoscopy or had low-risk lesions on endoscopy, and of these, 49.8% received continuous IV PPI. In patients with low-risk lesions, there was a 1.9-day difference in average length of stay between patients who received continuous IV PPI and intermittent IV PPI with no differences in outcome, and in patients who did receive endoscopy, there was a 2.6-day average LOS difference between the groups. In total, these patients with low-risk endoscopy lesions accounted for 3852.1 days of potentially unneeded hospitalization and potentially CAD 3.8 million/year in savings with intermittent PPI use.

There are a number of areas of controversy in the use of PPIs in hemostasis. The data are mixed on whether continuous IV PPI after endoscopic hemostasis reduces mortality [10,24]. The optimal dose and route of administration for post-endoscopy PPI therapy is unclear from the literature. Meta-analyses and systematic reviews are challenged by the heterogeneity of the definition of “high-dose” of PPI therapy [6,7,8,10,11,25,26]. Most studies define it as an IV bolus of 80 mg followed by an IV continuous infusion of 8 mg/hour for 72 h, totaling 656 mg IV over 72 h. Others used IV 40 mg every 12 h, for a total of 240 mg IV over 72 h [27,28]. One study used an IV 80 mg bolus followed by IV 40 mg every 6 h, for a total of 560 mg over 72 h [29]. Some studies used esomeprazole 40 mg PO every 12 h or rabeprazole 20 mg every 12 h and termed this “high-dose oral” therapy [30,31].

For patients with high-risk stigmata who received endoscopic hemostasis, the data support using continuous IV PPI therapy for 72 h post-endoscopy [10,11,25,26]. There are also data to suggest that smaller doses of PPI therapy, given intermittently, delivered either IV or PO, can have similar effects [6,7,8]. The level of evidence is not strong, leading to different recommendations when interpreting the same data, but it is certainly strong enough that no guidelines recommend against smaller doses of PPI [10,11,25,26]. Thus, if we are being conservative with our line of reasoning, low-risk patients who received no endoscopic hemostasis or no endoscopy at all should have no adverse events with IV intermittent or PO intermittent PPI therapy at higher-than-standard doses for NV-UGIB. In fact, the guidelines recommend PO standard dose (i.e., once daily) for this patient group [10,11,25,26].

Among the current guidelines (Table 3) for high-risk stigmata lesions treated with endoscopic hemostasis or adherent clots without hemostasis, both the American College of Gastroenterology (ACG) in 2021 and European Society of Gastrointestinal Endoscopy (ESGE) in 2021 recommended high-dose PPI (defined by the ACG as ≥80 mg/24 h) given either by continuous IV, intermittent IV, or intermittent PO for 72 h post-endoscopy [11,32]. The International Consensus Group in 2019 recommended an IV bolus and then continuous PPI for 72 h post-endoscopy [10], and the Asia-Pacific working group in 2018 recommended high-dose oral PPI as an adjunct [25]. For post-endoscopic intermittent IV PPI use (non high-dose), the International Consensus Group could not make a recommendation for or against [10], and the ESGE recommended considering intermittent IV dosing or high-dose oral PPI therapy [32]. For pre-endoscopic PPI, ACG guidelines could not make a recommendation for or against its use; the International Consensus Group did not comment on it in 2019 (previously recommended considering continuous IV PPI in 2010) [1,10]; the Asia-Pacific working Group did not agree to recommend it; and the ESGE recommended considering continuous IV PPI [10,11,25,32]. Based on our data, we would suggest starting with either continuous or intermittent IV PPI and switch to intermittent oral dosing if the lesions are low risk endoscopically.

Previous data from Canada, the United States, and United Kingdom have shown that there are often inappropriate indications for PPI use in hospital [17,33,34]. Pre-endoscopy, most patients in Canada receive appropriate IV PPI therapy, especially given the diagnostic uncertainly at times [33]. Unfortunately, the majority of patients who have had endoscopy and do not have an indication for IV continuous PPI therapy are still continued on it inappropriately, with national rates ranging from 56.9% to 91.5% [33,35] and local rates in Calgary being 47% [36]. Our finding of 45% patients receiving IV continuous PPI despite having no endoscopy or low-risk findings are similar to previous local data from a different period although lower than the nationally reported rates.

The previous literature on the cost of PPI use have looked at either regimens of IV PPI prior to endoscopy or PPI selection post-endoscopy. Prior to endoscopy, using continuous IV PPI has been shown to reduce the rate of endoscopic interventions [37] and reduce the risk of GI bleeding with incremental cost-effectiveness ratios of CAD 19,832 CDN/GI bleed averted [15]. After endoscopy, the literature to date is less clear. In a decision analysis model from a managed care organization perspective, the use of IV PPI as compared to oral PPI post endoscopy cost USD 708,735 per quality adjusted life year [38]. A second decision analytic model analyzed a spectrum of NV-UGIB and performed a budget impact analysis comparing multiple PPI strategies pre- and post-endoscopy. High-dose PPI pre- and post-endoscopy cost USD 11,399 in 2014. Different IV PPI dosing, whether continuous or intermittent, had a very modest effect on the total cost [16]. We similarly conclude that the formulation of the PPI itself represents a small percentage of the total hospitalization cost but that there is a signal towards decreased LOS if patients are not on a continuous IV PPI regimen post-endoscopy. A lower length of stay will have the highest impact on the cost of the total admission due to daily hospital charges as well as physician fees. In contrast to this model, our study offers real world admission data regarding length of stay that is risk-stratified, and we feel that it may provide cost data that are more reflective of reality.

Potassium-competitive acid blockers (P-CABs) are a new class of medications used for similar indications as PPIs (e.g., *Helicobacter pylori* treatment and peptic ulcer treatment). The first molecule in this class to be available clinically outside of trials was vonoprazan in Japan in 2015 [39]. As of mid-2023, there are no published manuscripts specifically addressing the use of P-CABs in NV-UGIB. A nationwide database study in Japan showed non-inferiority of vonoprazan compared to PPIs in rates of NV-UGIB in patients with ischemic heart disease receiving multiple anti-thrombotic medications [40]. There are multiple studies that comment on the effect of vonoprazan compared to PPIs for iatrogenic gastric or duodenal ulcers as a result of endoscopic submucosal dissection, with most finding an advantage with vonoprazan [41,42]. Finally, one study using Japanese pricing and a Markov simulation model found vonoprazan to be more cost-effective compared to lansoprazole or esomeprazole in quality-adjusted life years gained for patients taking low-dose acetylsalicylic acid for secondary prevention of cardiovascular diseases [43]. Thus, future updates on this topic may reflect on a bigger role in the NV-UGIB setting for P-CABs. These medications are currently not available in Canada and are only available in the United States as a part of a pre-packaged combination medication for the treatment of *H. pylori*.

### Limitations

The LOS differences generally do not reach significance in our subgroups, especially because of the low numbers of patients receiving intermittent IV PPI. Furthermore, we can only infer correlation between PPI administration and LOS through this administrative database. This could be an indirect effect from earlier ambulation (without an infusing IV pump), fewer falls from tripping on IV lines, or faster step-down to PO PPI therapy. Comparing the clinical parameters we collected, the patients receiving continuous IV PPI appear to be more ill, and this likely reflects physician comfort and clinical judgement. Truly establishing clinical difference or equivalence in these study populations would require a more intentional analysis of comorbidities which is not available in this dataset. Data regarding rebleeding, surgery, and mortality unfortunately are not captured in our database. Additionally, it is possible that some patients presenting with an upper GI bleed may have been missed if an order set were not used. In the authors’ experience, order sets are generally used with the initial presentation to hospital. As records from this administrative dataset were de-identified, there is limited information about comorbidities and medications available. As such, additional risk factors for UGI bleeding cannot be accounted for in this analysis.

The group of patients who had ICD codes of NV-UGIB yet did not have endoscopy show the limits of using administrative data to infer real-world situations. It is unclear whether these patients declined EGD, were too ill to undergo EGD, did not have EGD in their goals of care, or were simply miscoded. The CMG+ are limited in their ability to capture all the healthcare expenditure associated with a condition. Only half of patients coded as NVUGIB ended up undergoing EGD. Of those not receiving EGD, some received lower endoscopy instead. This speaks to the accuracy of ICD coding (the codes K92.0, K91.0, and K92.0 can have overlap from lower GI bleed as well).

Finally, we have grouped the patients who presented with NV-UGIB but did not receive endoscopy with other low-risk patients. There may be a subset of these patients that did not receive endoscopy because they were too ill and thus the PPI therapy was a “best non-invasive treatment” approach. We believe these patients constitute a smaller portion of this overall group, since the clinical characteristics were still overall less acute than the high-risk patient group.

## 5. Conclusions

In summary, our study uses large-number, local, real-world data to calculate potential cost savings from a minor medication administration change. With some basic assumptions about the pharmacodynamics and efficacy of PPIs and patient stratification, we can identify a population of patients that can safely receive intermittent IV PPI rather than continuous IV PPI. In a lean healthcare setting, this gives us further direction on quality improvement projects and advocacy in guidelines.

## Figures and Tables

**Table 1:** Demographic features stratified by risk of bleeding and treatment.

Variable	High-Risk Lesion					Low-Risk Lesion					No EGD				
	All	No PPI	PPI B + I	PPI I–IV	PO	All	No PPI	PPI B + I	PPI I–IV	PO	All	No PPI	PPI B + I	PPI I–IV	PO
Visits	410	36	337	12	25	1403	152	1079	60	112	2241	1373	582	61	225
No. (%) Female	133 (32)	10 (28)	110 (33)	4 (33)	9 (36)	553 (39)	72 (47)	406 (38)	26 (43)	49 (43)	1051 (47)	677 (49)	247 (42)	23 (38)	104 (46)
Mean Age (95% CI)	64 (62–66)	64 (59–69)	64 (62–66)	66 (54–78)	68 (62–73)	63 (63–65)	59 (56–62)	64 (62–65)	67 (63–72)	67 (64–71)	57 (56–58)	54 (53–56)	63 (61–65)	57 (51–63)	59 (56–62)
Age Range	19–66	26–95	19–96	31–93	44–91	18–103	18–97	18–103	20–94	21–100	18–106	18–101	19–106	19–98	19–96
Heart rate, mean, BPM (95% CI)	92 (91–94)	89 (83–95)	93 (91–95)	98 (88–108)	87 (79–95)	91 (90–92)	86 (83–89)	92 (91–93)	89 (84–95)	85 (82–88)	88 (87–89)	86 (85–87)	93 (91–95)	93 (87–98)	87 (84–89)
Systolic Blood pressure, mean, mm Hg (95% CI)	119 (116–121)	126 (119–132)	117 (115–119)	131 (117–144)	126 (117–134)	126 (125–128)	132 (129–135)	125 (124–127)	127 (121–133)	128 (124–132)	132 (131–133)	134 (133–135)	128 (126–130)	131 (126–137)	131 (128–134)
Diastolic Blood pressure, mean, mm Hg (95% CI)	69 (67–71)	75 (68–81)	68 (67–70)	76 (69–82)	68 (63–74)	73 (72–75)	77 (74–79)	73 (71–75)	74 (70–78)	70 (67–73)	78 (77–79)	80 (79–80)	76 (73–78)	75 (70–79)	76 (74–78)
Hemoglobin, mean, g/L (95% CI)	93 (90–95)	108 (97–118)	91 (88–94)	103 (85–122)	91 (80–102)	103 (101–104)	117 (112–122)	101 (99–103)	105 (96–113)	100 (95–106)	125 (124–127)	132 (130–133)	114 (111–116)	119 (111–127)	123 (119–127)
Number transfused (%)	195 (48)	5 (14)	174 (52)	4 (33)	12 (48)	476 (34)	20 (13)	411 (38)	16 (27)	29 (26)	249 (11)	71 (5)	139 (24)	7 (11)	32 (14)
Number admitted (%)	392 (96)	29 (81)	327 (97)	12 (100)	24 (96)	1191 (85)	98 (64)	942 (87)	52 (87)	99 (88)	746 (33)	216 (16)	383 (66)	27 (44)	120 (53)

B + I = bolus and infusion, I–IV = intermittent intravenous, PO = per os (by mouth), PPI = proton pump inhibitor.

**Table 2:** Estimated costs of admission. All dollar amounts are in 2017 Canadian dollars.

	Visits	Average LOS(95% CI)	Average Cost Per Admission						Overall Costs
			Total Cost	Lower 95%	Upper 95%	Hospital Cost	Physician Cost	Drug Cost	
High Risk	410	7.4 (6.3–8.4)	N/A						
no PPI	36	5.2 (3.5–7)	CAD 7514	CAD 5155	CAD 10,011	CAD 6673	CAD 841	CAD 0	CAD 270,491
bolus + infusion	337	7.5 (6.3–8.8)	CAD 10,742	CAD 9077	CAD 12,545	CAD 9624	CAD 1860	CAD 38	CAD 3,620,086
intermittent IV	12	11.6 (5.8–17.3)	CAD 16,408	CAD 8363	CAD 24,316	CAD 14,885	CAD 2713	CAD 17	CAD 196,901
PO	25	5.8 (3.5–8.2)	CAD 8348	CAD 5158	CAD 11,678	CAD 7443	CAD 1507	CAD 2	CAD 208,707
Subgroup Total									CAD 4,296,185
Low Risk	1403	6.6 (6.1–7.2)	N/A						
no PPI	152	4.2 (3.4–4.9)	CAD 6126	CAD 5017	CAD 7097	CAD 5389	CAD 737	CAD 0	CAD 931,216
bolus + infusion	1231	6.8 (6.2–7.3)	CAD 9771	CAD 8938	CAD 10,464	CAD 8726	CAD 1007	CAD 38	CAD 10,542,659
intermittent IV	60	4.9 (3.9–5.9)	CAD 7111	CAD 5724	CAD 8499	CAD 6288	CAD 810	CAD 14	CAD 426,686
PO	112	6.3 (4.8–7.8)	CAD 9042	CAD 6961	CAD 11,123	CAD 8084	CAD 955	CAD 3	CAD 1,012,713
Subgroup Total									CAD 12,913,274
No EGD	2241	7.9 (7.5–8.4)	N/A						
no PPI	1373	4.4 (4.1–4.6)	CAD 6291	CAD 5875	CAD 6568	CAD 5646	CAD 645	CAD 0	CAD 8,637,095
bolus + infusion	582	11.1 (9.7–12.7)	CAD 15,624	CAD 13,682	CAD 17,705	CAD 14,244	CAD 1341	CAD 39	CAD 9,093,343
intermittent IV	61	8.5 (6–11)	CAD 11,994	CAD 8526	CAD 15,462	CAD 10,907	CAD 1071	CAD 15	CAD 731,612
PO	225	7.7 (6.5–8.9)	CAD 10,872	CAD 9207	CAD 12,536	CAD 9881	CAD 988	CAD 3	CAD 2,446,098
Subgroup Total									CAD 20,908,148
Grand Total									CAD 38,117,607

**Table 3:** Summary of current guidelines on PPI use for NV-UGB.

	American College of Gastroenterology (ACG) (2021) [11]	International Consensus Group (2019) [10]	Asia-Pacific Working Group (2018) [25]	European Society of Gastrointestinal Endoscopy (ESGE) (2021) [32]
Post-endoscopy finding high-risk lesion	IV bolus + infusion	IV bolus + infusion	High-dose PO PPI as adjunct	IV bolus + infusion
	High-dose intermittent, IV or PO	Neither for nor against intermittent IV	No comment on intermittent IV	Can consider intermittent IV or high-dose PO
Pre-endoscopy	No recommendation. Previously recommended considering IV bolus + infusion	No comment Previously recommended IV bolus + infusion in 2010 version of guidelines	No agreement on recommending PPI	Consider IV bolus + infusion

## Data Availability

The data presented in this study are available on request from the corresponding author.

## Supplementary Materials

The following supporting information can be downloaded at: https://www.mdpi.com/article/10.3390/medicines10070044/s1, Table S1: ICD-10 Codes Used for Data Extraction; Table S2: Canadian Classification of Health Intervention Codes Used for Stratifying Patients.
