# Supplementary material for: Intermittent Proton Pump Inhibitor Therapy in Low-Risk Non-Variceal Upper Gastrointestinal Bleeding May Be Significantly Cost-Saving
Source: Medicines (Basel). 2023 Jul 20;10(7):44. doi: 10.3390/medicines10070044 (PMC10384205; doi:10.3390/medicines10070044)
Supplement: Supplementary file 1 [file medicines-10-00044-s001.zip › medicines-2463278-supplementary.pdf]

Table S1. ICD-10 Codes Used for Data Extraction.

ICD-10 Codes		Dataset	Quan[1]	Valkhoff [2]	Ahmed[3]
K226	Gastro-oesophageal laceration-haemorrhage syndrome	K226			K226
K250	Gastric ulcer, acute with haemorrhage	K250	K25.0	K25.0	K250
K251	Gastric ulcer, acute with perforation			K25.1	
K252	Gastric ulcer, acute with both haemorrhage and perforation		K25.2	K25.2	K252
K253	Gastric ulcer, acute without haemorrhage or perforation				
K254	Gastric ulcer, chronic or unspecified with haemorrhage	K254	K25.4		K254
K255	Gastric ulcer, chronic or unspecified with perforation				
K256	Gastric ulcer, chronic or unspecified with both haemorrhage and perforation	K256	K25.6		K256
K257	Gastric ulcer, chronic with haemorrhage or perforation				
K259	Gastric ulcer, unspecified as acute or chronic, without haemorrhage or perforation				
K260	Duodenal ulcer, acute with haemorrhage	K260	K26.0	K26.0	K260
K261	Duodenal ulcer, acute with perforation			K26.1	
K262	Duodenal ulcer, acute with both haemorrhage and perforation		K26.2	K26.2	K262
K263	Duodenal ulcer, acute without haemorrhage or perforation				
K264	Duodenal ulcer, chronic or unspecified with haemorrhage	K264	K26.4		K264
K265	Duodenal ulcer, chronic or unspecified with perforation				
K266	Duodenal ulcer, chronic or unspecified with both haemorrhage and perforation	K266	K26.6		K266
K267	Duodenal ulcer, chronic with haemorrhage or perforation				
K269	Duodenal ulcer, unspecified as acute or chronic, without haemorrhage or perforation				
K270	Peptic ulcer, acute with haemorrhage	K270	K27.0	K27.0	K270
K271	Peptic ulcer, acute with perforation			K27.1	
K272	Peptic ulcer, acute with both haemorrhage and perforation		K27.2	K27.2	K272
K273	Peptic ulcer, acute without haemorrhage or perforation				
K274	Peptic ulcer, chronic or unspecified with haemorrhage	K274	K27.4		K274
K275	Peptic ulcer, chronic or unspecified with perforation				

K276	Peptic ulcer, chronic or unspecified with both haemorrhage and perforation		K27.6		K276
K277	Peptic ulcer, chronic with haemorrhage or perforation				
K279	Peptic ulcer, unspecified as acute or chronic, without haemorrhage or perforation				
K280	Gastrojejunal ulcer, acute with haemorrhage		K28.0	K28.0	K280
K281	Gastrojejunal ulcer, acute with perforation			K28.1	
K282	Gastrojejunal ulcer, acute with both haemorrhage and perforation		K28.2	K28.2	K282
K283	Gastrojejunal ulcer, acute without haemorrhage or perforation				
K284	Gastrojejunal ulcer, chronic or unspecified with haemorrhage	K284	K28.4		K284
K285	Gastrojejunal ulcer, chronic or unspecified with perforation				
K286	Gastrojejunal ulcer, chronic or unspecified with both haemorrhage and perforation		K28.6		K286
K287	Gastrojejunal ulcer, chronic with haemorrhage or perforation				
K289	Gastrojejunal ulcer, unspecified as acute or chronic, without haemorrhage or perforation				
K290	Acute haemorrhagic gastritis			K29.0	K290
K920	Haematemesis	K920			K920
K921	Melaena	K921		K92.1	K921
K922	Gastrointestinal haemorrhage, unspecified	K922		K92.2	K922

1. Quan S. Upper-gastrointestinal bleeding secondary to peptic ulcer disease: Incidence and outcomes. *WJG*. 2014;20(46):17568. doi:10.3748/wjg.v20.i46.17568

2. Valkhoff VE, Coloma PM, Masclee GMC, et al. Validation study in four health-care databases: upper gastrointestinal bleeding misclassification affects precision but not magnitude of drug-related upper gastrointestinal bleeding risk. *Journal of Clinical Epidemiology*. 2014;67(8):921-931. doi:10.1016/j.jclinepi.2014.02.020

3. Ahmed A. Upper gastrointestinal bleeding in Scotland 2000-2010: Improved outcomes but a significant weekend effect. *WJG*. 2015;21(38):10890. doi:10.3748/wjg.v21.i38.10890

Table S2. Canadian Classification of Health Intervention Codes Used for Stratifying Patients.

Procedure Classification	Canadian Classification of Health Interventions Codes
**High risk**	1NA13BAGX
	1NA13BAX7
	1NA13DAE3
	1NF13BABD
	1NF13BAC2
	1NF13BAGX
	1NF13BAKK
	1NF13BAW4
	1NF13BAX7
	1NF13GPGE
	1NP13BAC2
	1NP13BAGX
	1NP13BAKK
	1NP13BAX7
	1NP13GQGE
**Low risk**	2NA71BA
	2NK71BABJ
	2NK71BABL
	2NF71BA
	2NA70BA
	2NK70BCBK
	2NK70BABJ
	2NK70BABL
	2NK70BDBK
	2NF70BA

High risk procedures were considered to have codes related to control of bleeding or interventional radiology procedures. Low risk procedures were identified by codes related to inspection or biopsy of the esophagus, stomach or small bowel.
